# 4,4′-(Ethene-1,2-di­yl)dipyridinium 4-[2-(pyridin-4-yl)ethen­yl]pyridinium octa­cyanidomolybdate(V) tetra­hydrate

**DOI:** 10.1107/S1600536813002985

**Published:** 2013-02-02

**Authors:** Xiao-Zhen Yang, Ai-Yun Hu, Ai-Hua Yuan

**Affiliations:** a School of Material Science and Engineering, Jiangsu University of Science and Technology, Zhenjiang 212003, People’s Republic of China; b School of Biology and Chemical Engineering, Jiangsu University of Science and Technology, Zhenjiang 212003, People’s Republic of China

## Abstract

The crystal structure of the title compound, (C_12_H_12_N_2_)(C_12_H_11_N_2_)[Mo(CN)_8_]·4H_2_O, consists of 4,4′-(ethene-1,2-di­yl)dipyridinium and 4-[2-(pyridin-4-yl)ethen­yl]pyridinium cations disordered over the same site, an [Mo(CN)_8_]^3−^ anion and four water mol­ecules of crystallization. The eight-coordinate [Mo(CN)_8_]^3−^ unit exhibits a slightly distorted square-anti­prismatic geometry. In the structure, the cations (crystallographic symmetry, 2) and anions (crystallographic symmetry, 222) are arranged alternately by N—H⋯O and O—H⋯N hydrogen bonds, forming layers parallel to the *bc* plane. These layers are further linked through O—H⋯N hydrogen bonds, generating a three-dimensional supra­molecular network.

## Related literature

For general background to the design and construction of multi-functional materials, see: Nowicka *et al.* (2012 ▶); Prins *et al.* (2007 ▶); Sieklucka *et al.* (2011 ▶); Tanase *et al.* (2008 ▶); Zhou *et al.* (2012 ▶). For related structures, see: Liu *et al.* (2008 ▶); Qian *et al.* (2009 ▶).
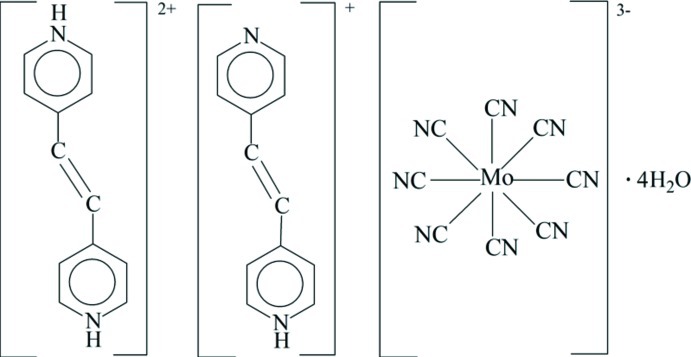


## Experimental

### 

#### Crystal data

(C_12_H_12_N_2_)(C_12_H_11_N_2_)[Mo(CN)_8_]·4H_2_O
*M* *_r_* = 743.63
Orthorhombic, 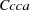

*a* = 12.403 (3) Å
*b* = 16.534 (3) Å
*c* = 15.370 (3) Å
*V* = 3151.8 (11) Å^3^
*Z* = 4
Mo *K*α radiation
μ = 0.48 mm^−1^
*T* = 291 K
0.18 × 0.15 × 0.13 mm

#### Data collection

Bruker SMART APEXII diffractometer
Absorption correction: multi-scan (*SADABS*; Bruker, 2004 ▶) *T*_min_ = 0.919, *T*_max_ = 0.941
6789 measured reflections
1442 independent reflections
1388 reflections with *I* > 2σ(*I*)
*R*_int_ = 0.016

#### Refinement

*R*[*F* ^2^ > 2σ(*F* ^2^)] = 0.021
*wR*(*F* ^2^) = 0.052
*S* = 1.09
1442 reflections
112 parameters
H-atom parameters constrained
Δρ_max_ = 0.28 e Å^−3^
Δρ_min_ = −0.31 e Å^−3^

### 

Data collection: *APEX2* (Bruker, 2004 ▶); cell refinement: *SAINT* (Bruker, 2004 ▶); data reduction: *SAINT*; program(s) used to solve structure: *SHELXS97* (Sheldrick, 2008 ▶); program(s) used to refine structure: *SHELXL97* (Sheldrick, 2008 ▶); molecular graphics: *DIAMOND* (Brandenburg, 2006 ▶); software used to prepare material for publication: *SHELXTL* (Sheldrick, 2008 ▶).

## Supplementary Material

Crystal structure: contains datablock(s) I, global. DOI: 10.1107/S1600536813002985/rz5039sup1.cif

Structure factors: contains datablock(s) I. DOI: 10.1107/S1600536813002985/rz5039Isup2.hkl

Additional supplementary materials:  crystallographic information; 3D view; checkCIF report

## Figures and Tables

**Table 1:** Hydrogen-bond geometry (Å, °)

*D*—H⋯*A*	*D*—H	H⋯*A*	*D*⋯*A*	*D*—H⋯*A*
O1—H1*A*⋯N1^i^	0.85	2.11	2.9524 (19)	174
O1—H1*B*⋯N2^ii^	0.85	2.00	2.8195 (18)	162
N3—H3*X*⋯O1	0.89	1.86	2.7342 (17)	166

Symmetry codes: (i) 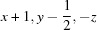; (ii) 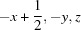.

**Fig. 1.**
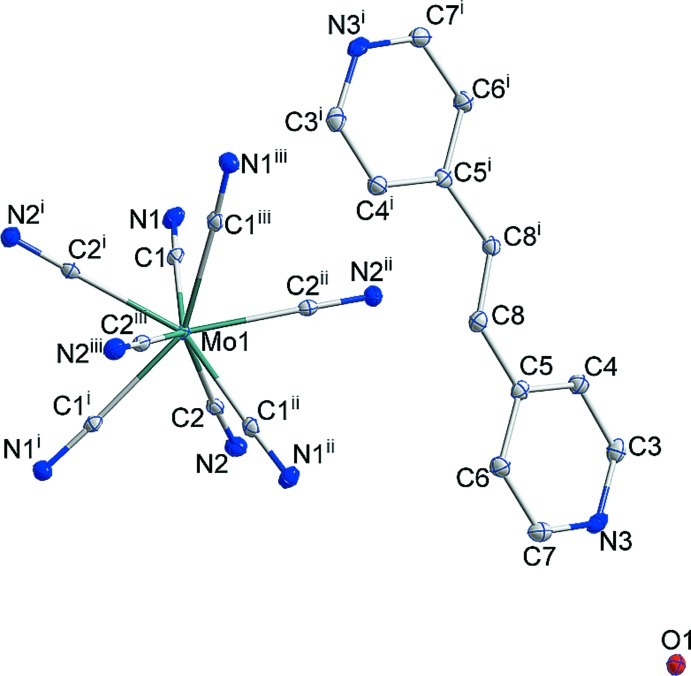
The molecular structure of the title compound with thermal ellipsoids at the 30% probability level. All H atoms were removed for clarity. Symmetry codes: (i) x, -y + 1/2, -z + 1/2; (ii) -x, -y + 1/2, z; (iii) -x, y, -z + 1/2

**Fig. 2.**
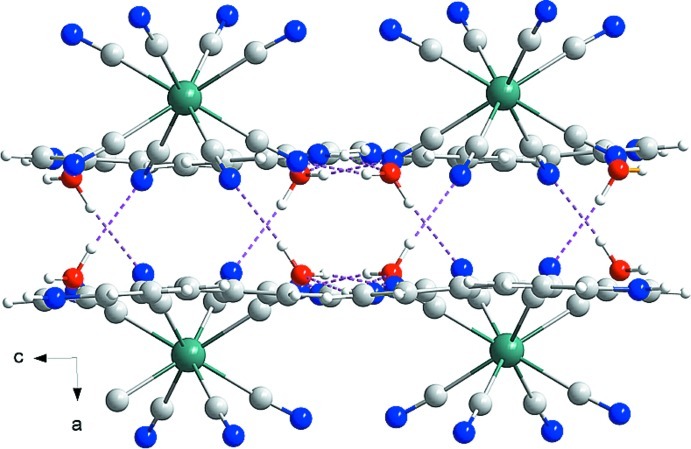
The three-dimensional supramolecular network of the title compound.

## supplementary crystallographic information

### Comment

In the past few years, much attention has been put into the design and construction of multi-functional materials (Zhou *et al.*, 2012). Octacyanometallates [*M*(CN)_8_]*^n^*^-^ (*M* = Mo, W; *n* = 3, 4) with flexible coordination modes and lower symmetries have been aggressively studied recently (Nowicka *et al.*, 2012), because these building blocks can adopt various geometries, *e.g.*, square antiprismatic, dodecahedral or bicapped trigonal prismatic, depending on the external environments. The combination of the [*M*(CN)_8_]*^n^*^-^ precusors and the second metal centers has produced various dimensional molecular structures and the resulting materials have displayed intriguing properties (Sieklucka *et al.*, 2011). However, the development of octacyano- and lanthanide-based assemblies has been somewhat hampered by the tendency of the lanthanide ions to adopt higher coordination numbers, their ability to easily adapt to a given environment, and in the absence of design strategies for 4f-4d/5d networks. Recently, we used [Mo^V^(CN)_8_]^3-^ as building block to react with the lanthanide ion Ce^3+^ and dpe ligand (dpe = 1,2-di(pyridin-4-yl)ethylene), in order to obtain new octacyanide-based 4f/4 d compound with open structure. Unfortunately, a new ion-pair compound without Ce^3+^ ions was isolated. The asymmetric unit of the title compound contains 4,4'-ethene-1,2-diyldipyridinium, [H_2_dpe]^2+^, and 4-(2-(pyridin-4-yl)ethenyl)pyridinium, [Hdpe]^+^, cations, one [Mo(CN)_8_]^3-^ anion, and four crystallized water molecules (Fig. 1). Both the [H_2_dpe]^2+^ and [Hdpe]^+^ cations are disordered over the same site. The eight-coordinated [Mo(CN)_8_] unit exhibits a distorted slightly square antiprismatic geometry, typical of octacyanometalates (Prins *et al.*, 2007; Tanase *et al.*, 2008). The average distances of Mo1—C and C—N bonds are 2.1682 and 1.156 Å, respectively, while the Mo1—CN bonds are almost linear with the maximum deviation from linearity of 3.8°.

In the structure, [H_2_dpe]^2+^ cation, [Hdpe]^+^ cation and [Mo(CN)_8_]^3-^ unit are arranged alternatively through N3—H3X···O1 and O1—H1B···N2^v^ (symmetric code: (v) -*x* + 1/2, -*y*, *z*) hydrogen bonds (Table 1) to generate a two-dimensional layer. These layers are further interlinked through O1—H1A···N1^iv^ (symmetric code: (iv) *x* + 1, *y* - 1/2, -*z*) hydrogen bonds, forming a three-dimensional supramolecular network (Fig. 2). This structural feature has also been observed in related octacyanide-based compounds (C_10_H_10_N_4_)(C_10_H_9_N_4_)[*M*(CN)_8_].*n*H_2_O (*M* = Mo, W) (Qian *et al.*, 2009; Liu *et al.*, 2008).

### Experimental

Single crystals of the title compound were prepared at room temperature by slow diffusion of a CH_3_CN/H_2_O (1:1 *v*/*v*) solution containing both Ce^III^(NO_3_)_3_.6H_2_O (0.05 mmol) and dpe (0.15 mmol) in a CH_3_CN/H_2_O (1:1 *v*/*v*) solution of [HN(*n*-C_4_H_9_)_3_]_3_[Mo^V^(CN)_8_].4H_2_O (0.05 mmol). After four weeks, dark-blue rod-like crystals were obtained.

### Refinement

All non-H atoms were refined anisotropically. The (C)H atoms were calculated at idealized positions and included in the refinement in a riding mode. The (N)H and (O)H atoms of water molecules were located from a difference Fourier map and refined as riding [N—H = 0.89 Å, *U*(H) = 1.2*U*_eq_(N); O—H = 0.85 Å, *U*(H) = 1.5*U*_eq_(O)], with the occupancy factor of the N-bound H atoms set to. 0.75

### Crystal data

**Table:**

(C_12_H_12_N_2_)(C_12_H_11_N_2_)[Mo(CN)_8_]·4H_2_O	*F*(000) = 1524
*M**_r_* = 743.63	*D*_x_ = 1.567 Mg m^−^^3^
Orthorhombic, *C**c**c**a*	Mo *K*α radiation, λ = 0.71073 Å
Hall symbol: -C 2b 2bc	Cell parameters from 6602 reflections
*a* = 12.403 (3) Å	θ = 3.4–29.0°
*b* = 16.534 (3) Å	µ = 0.48 mm^−^^1^
*c* = 15.370 (3) Å	*T* = 291 K
*V* = 3151.8 (11) Å^3^	Rod, dark blue
*Z* = 4	0.18 × 0.15 × 0.13 mm

### Data collection

**Table:**

Bruker SMART APEXII diffractometer	1442 independent reflections
Radiation source: fine-focus sealed tube	1388 reflections with *I* > 2σ(*I*)
Graphite monochromator	*R*_int_ = 0.016
phi and ω scans	θ_max_ = 25.3°, θ_min_ = 3.4°
Absorption correction: multi-scan (*SADABS*; Bruker, 2004)	*h* = −14→14
*T*_min_ = 0.919, *T*_max_ = 0.941	*k* = −15→19
6789 measured reflections	*l* = −18→16

### Refinement

**Table:**

Refinement on *F*^2^	Primary atom site location: structure-invariant direct methods
Least-squares matrix: full	Secondary atom site location: difference Fourier map
*R*[*F*^2^ > 2σ(*F*^2^)] = 0.021	Hydrogen site location: inferred from neighbouring sites
*wR*(*F*^2^) = 0.052	H-atom parameters constrained
*S* = 1.09	*w* = 1/[σ^2^(*F*_o_^2^) + (0.0237*P*)^2^ + 4.1639*P*] where *P* = (*F*_o_^2^ + 2*F*_c_^2^)/3
1442 reflections	(Δ/σ)_max_ < 0.001
112 parameters	Δρ_max_ = 0.28 e Å^−^^3^
0 restraints	Δρ_min_ = −0.31 e Å^−^^3^

### Special details

**Table:**

Geometry. All e.s.d.'s (except the e.s.d. in the dihedral angle between two l.s. planes) are estimated using the full covariance matrix. The cell e.s.d.'s are taken into account individually in the estimation of e.s.d.'s in distances, angles and torsion angles; correlations between e.s.d.'s in cell parameters are only used when they are defined by crystal symmetry. An approximate (isotropic) treatment of cell e.s.d.'s is used for estimating e.s.d.'s involving l.s. planes.
Refinement. Refinement of *F*^2^ against ALL reflections. The weighted *R*-factor *wR* and goodness of fit *S* are based on *F*^2^, conventional *R*-factors *R* are based on *F*, with *F* set to zero for negative *F*^2^. The threshold expression of *F*^2^ > σ(*F*^2^) is used only for calculating *R*-factors(gt) *etc*. and is not relevant to the choice of reflections for refinement. *R*-factors based on *F*^2^ are statistically about twice as large as those based on *F*, and *R*- factors based on ALL data will be even larger.

### Fractional atomic coordinates and isotropic or equivalent isotropic displacement parameters (Å^2^)

**Table:**

	*x*	*y*	*z*	*U*_iso_*/*U*_eq_	Occ. (<1)
Mo1	0.0000	0.2500	0.2500	0.01026 (10)	
O1	0.65008 (10)	−0.07739 (7)	−0.07004 (7)	0.0222 (3)	
H1A	0.7081	−0.0819	−0.0991	0.033*	
H1B	0.6515	−0.1156	−0.0330	0.033*	
N1	−0.15776 (12)	0.39926 (8)	0.18061 (8)	0.0217 (3)	
N2	−0.12604 (11)	0.18087 (8)	0.07447 (9)	0.0214 (3)	
N3	0.61502 (11)	0.04861 (9)	0.04165 (9)	0.0222 (3)	
H3X	0.6177	0.0114	−0.0001	0.027*	0.75
C1	−0.10315 (12)	0.34816 (9)	0.20714 (9)	0.0153 (3)	
C2	−0.08556 (13)	0.20481 (9)	0.13705 (10)	0.0153 (3)	
C3	0.61146 (13)	0.12818 (10)	0.02559 (10)	0.0226 (4)	
H3	0.6063	0.1466	−0.0314	0.027*	
C4	0.61534 (13)	0.18237 (10)	0.09245 (10)	0.0197 (3)	
H4	0.6125	0.2375	0.0809	0.024*	
C5	0.62366 (12)	0.15508 (9)	0.17825 (10)	0.0171 (3)	
C6	0.62332 (13)	0.07142 (10)	0.19249 (11)	0.0212 (4)	
H6	0.6258	0.0511	0.2489	0.025*	
C7	0.61929 (14)	0.01956 (10)	0.12305 (11)	0.0239 (4)	
H7	0.6195	−0.0360	0.1324	0.029*	
C8	0.62950 (13)	0.20979 (10)	0.25268 (10)	0.0177 (3)	
H8	0.6335	0.1868	0.3078	0.021*	

### Atomic displacement parameters (Å^2^)

**Table:**

	*U*^11^	*U*^22^	*U*^33^	*U*^12^	*U*^13^	*U*^23^
Mo1	0.01259 (15)	0.00997 (14)	0.00822 (14)	0.000	0.000	0.000
O1	0.0281 (6)	0.0209 (6)	0.0175 (6)	0.0004 (5)	0.0046 (5)	−0.0005 (5)
N1	0.0279 (8)	0.0213 (7)	0.0158 (7)	0.0060 (6)	−0.0014 (6)	−0.0007 (6)
N2	0.0263 (8)	0.0205 (7)	0.0175 (7)	−0.0032 (6)	−0.0032 (6)	−0.0003 (6)
N3	0.0209 (7)	0.0244 (8)	0.0213 (7)	0.0016 (6)	0.0000 (6)	−0.0098 (6)
C1	0.0185 (8)	0.0168 (8)	0.0107 (7)	−0.0012 (6)	0.0010 (6)	−0.0029 (6)
C2	0.0168 (8)	0.0125 (8)	0.0166 (8)	−0.0007 (6)	0.0011 (7)	0.0027 (6)
C3	0.0219 (8)	0.0295 (10)	0.0164 (8)	0.0023 (7)	0.0000 (7)	−0.0004 (7)
C4	0.0222 (8)	0.0181 (8)	0.0188 (8)	0.0018 (6)	0.0005 (7)	0.0007 (6)
C5	0.0141 (7)	0.0187 (8)	0.0186 (8)	0.0013 (6)	0.0003 (6)	−0.0010 (6)
C6	0.0253 (9)	0.0193 (8)	0.0190 (8)	0.0005 (7)	−0.0006 (7)	0.0013 (6)
C7	0.0255 (9)	0.0182 (9)	0.0281 (9)	0.0005 (7)	−0.0011 (8)	−0.0028 (7)
C8	0.0184 (8)	0.0199 (8)	0.0149 (8)	0.0019 (7)	−0.0011 (6)	0.0002 (6)

### Geometric parameters (Å, º)

**Table:**

Mo1—C2	2.1674 (16)	N3—C7	1.341 (2)
Mo1—C2^i^	2.1674 (16)	N3—H3X	0.8896
Mo1—C2^ii^	2.1674 (16)	C3—C4	1.364 (2)
Mo1—C2^iii^	2.1674 (16)	C3—H3	0.9300
Mo1—C1^i^	2.1690 (16)	C4—C5	1.398 (2)
Mo1—C1^iii^	2.1690 (16)	C4—H4	0.9300
Mo1—C1	2.1690 (16)	C5—C6	1.400 (2)
Mo1—C1^ii^	2.1690 (16)	C5—C8	1.460 (2)
O1—H1A	0.8501	C6—C7	1.370 (2)
O1—H1B	0.8505	C6—H6	0.9300
N1—C1	1.157 (2)	C7—H7	0.9300
N2—C2	1.155 (2)	C8—C8^i^	1.332 (3)
N3—C3	1.339 (2)	C8—H8	0.9300
			
C2—Mo1—C2^i^	121.37 (8)	C1^iii^—Mo1—C1^ii^	107.72 (8)
C2—Mo1—C2^ii^	73.57 (8)	C1—Mo1—C1^ii^	144.64 (8)
C2^i^—Mo1—C2^ii^	139.67 (8)	H1A—O1—H1B	105.6
C2—Mo1—C2^iii^	139.67 (8)	C3—N3—C7	121.66 (14)
C2^i^—Mo1—C2^iii^	73.57 (8)	C3—N3—H3X	123.2
C2^ii^—Mo1—C2^iii^	121.37 (8)	C7—N3—H3X	115.1
C2—Mo1—C1^i^	72.33 (5)	N1—C1—Mo1	177.01 (13)
C2^i^—Mo1—C1^i^	74.09 (6)	N2—C2—Mo1	176.36 (14)
C2^ii^—Mo1—C1^i^	142.18 (5)	N3—C3—C4	120.35 (15)
C2^iii^—Mo1—C1^i^	77.74 (6)	N3—C3—H3	119.8
C2—Mo1—C1^iii^	142.18 (5)	C4—C3—H3	119.8
C2^i^—Mo1—C1^iii^	77.74 (6)	C3—C4—C5	120.08 (15)
C2^ii^—Mo1—C1^iii^	72.33 (5)	C3—C4—H4	120.0
C2^iii^—Mo1—C1^iii^	74.09 (6)	C5—C4—H4	120.0
C1^i^—Mo1—C1^iii^	144.64 (8)	C4—C5—C6	117.78 (14)
C2—Mo1—C1	74.09 (6)	C4—C5—C8	122.88 (14)
C2^i^—Mo1—C1	72.33 (5)	C6—C5—C8	119.32 (14)
C2^ii^—Mo1—C1	77.74 (6)	C7—C6—C5	119.78 (15)
C2^iii^—Mo1—C1	142.18 (5)	C7—C6—H6	120.1
C1^i^—Mo1—C1	107.72 (8)	C5—C6—H6	120.1
C1^iii^—Mo1—C1	83.12 (8)	N3—C7—C6	120.27 (15)
C2—Mo1—C1^ii^	77.74 (6)	N3—C7—H7	119.9
C2^i^—Mo1—C1^ii^	142.18 (5)	C6—C7—H7	119.9
C2^ii^—Mo1—C1^ii^	74.09 (6)	C8^i^—C8—C5	124.74 (18)
C2^iii^—Mo1—C1^ii^	72.33 (5)	C8^i^—C8—H8	117.6
C1^i^—Mo1—C1^ii^	83.12 (8)	C5—C8—H8	117.6

Symmetry codes: (i) *x*, −*y*+1/2, −*z*+1/2; (ii) −*x*, −*y*+1/2, *z*; (iii) −*x*, *y*, −*z*+1/2.

### Hydrogen-bond geometry (Å, º)

**Table:**

*D*—H···*A*	*D*—H	H···*A*	*D*···*A*	*D*—H···*A*
O1—H1*A*···N1^iv^	0.85	2.11	2.9524 (19)	174
O1—H1*B*···N2^v^	0.85	2.00	2.8195 (18)	162
N3—H3*X*···O1	0.89	1.86	2.7342 (17)	166

Symmetry codes: (iv) *x*+1, *y*−1/2, −*z*; (v) −*x*+1/2, −*y*, *z*.
